# Preventing Acute Limb Ischemia during VA-ECMO—In Silico Analysis of Physical Parameters Associated with Lower Limb Perfusion

**DOI:** 10.3390/jcm12186049

**Published:** 2023-09-19

**Authors:** Tifanie Brockaert, Inês Ferreira, Anne Laplante, Paul Fogel, David Grimbert, Pierre Mordant

**Affiliations:** 1 Université de Technologie de Compiègne, 60200 Compiègne, France; 2 Iperf, 1 Avenue de Champfleury, 69410 Champagne-au-Mont-d’Or, Franceanne.laplante@iperfconnecting.com (A.L.); 3 Mazars, Tour Exaltis 61 rue Henri Regnault, 92400 Courbevoie, France; pfogel@advestis.com (P.F.); david.grimbert@mazars.fr (D.G.); 4 Service de Chirurgie Vasculaire, Thoracique, et Transplantation Pulmonaire, Hôpital Bichat, Université Paris—Cité, Assistance Publique—Hôpitaux de Paris, 75018 Paris, France

**Keywords:** VA-ECMO, acute limb ischemia, distal perfusion catheter, CFD, arterial cannula

## Abstract

Background: Peripheral femoro-femoral veno-arterial extracorporeal membrane oxygenation is increasingly used in refractory cardiogenic shock. However, the obstruction of the femoral artery by the return cannula could lead to acute limb ischemia, a frequently encountered situation that is inconstantly prevented by the adjunction of a distal perfusion cannula (DPC). The aim of this study was to investigate the influence of three physical parameters on the perfusion of the cannulated lower limb. Methods: Using patient-specific arterial models and computational fluid dynamic simulations, we studied four diameters of arterial cannula, three diameters of DPC, and two percentages of arterial section limitation. Results: We found that adequate perfusion of the cannulated limb was achieved in only two out of the twenty-one configurations tested, specifically, when the arterial cannula had a diameter of 17 Fr, was considered to limit the section of the artery by 90%, and was associated with an 8 Fr or a 10 Fr DPC. Multivariable analysis revealed that the perfusion of the cannulated lower limb was correlated with the diameter of the DPC, but also with the diameter of the arterial cannula and the percentage of arterial section limitation. Conclusions: In most of the cases simulated here, the current system combining unsized arterial cannula and non-specific DPC was not sufficient to provide adequate perfusion of the cannulated lower limb, urging the need for innovative strategies to efficiently prevent acute limb ischemia during peripheral femoro-femoral veno-arterial extracorporeal membrane oxygenation.

## 1. Introduction

Veno-arterial extracorporeal membrane oxygenation (VA-ECMO) is an extracorporeal circulatory support system for the heart and lungs. It is notably used for patients with refractory cardiogenic shock secondary to cardiac surgery, decompensated cardiomyopathy, acute myocardial infarction, or refractory cardiac arrest [1]. VA-ECMO can be used as a bridge to heart or lung transplantation and as a bridge to organ recuperation after such transplants. During VA-ECMO, the patient’s deoxygenated blood is drained through a venous cannula, passed through an oxygenator, and reinjected through an arterial cannula. In adults, the cannulation is often done peripherally in the femoral vein and artery [1]. Current arterial cannulas allow good perfusion of the upper body and thus adequate circulatory support, but they can prevent blood flow from reaching the lower limb downstream of the cannula, which can lead to ipsilateral acute limb ischemia (ALI) [2]. Ipsilateral ALI occurs in 11 to 52% of VA-ECMO cases and can lead to further complications, including compartment syndrome requiring fasciotomy, functional deficit, or amputation of the lower limb. ALI carries a higher risk of a lethal outcome than other complications of VA-ECMO [3]. The burden of ALI might even counterbalance the physiological benefit of ECMO and hinder the survival benefit of this technique [4].

Some techniques have been developed to prevent ALI during VA-ECMO. The current gold standard is the placement of an ipsilateral antegrade distal perfusion cannula (DPC) of 5 to 10 Fr in the superficial femoral artery [5,6]. Current guidelines advocate the insertion of the DPC in advance, especially if the arterial cannula is larger than 19 Fr, as placing it after cannulation can be challenging and less efficient [6,7]. However, patients who need ECMO are often unstable, and the arterial cannula must be inserted as quickly as possible to establish efficient circulatory support [8]. It is then not always possible to place the DPC before the arterial cannulation. Furthermore, it is recommended to insert the DPC in the superficial femoral artery (SFA) [6,9], but the main channel that perfuses the lower limb in patients with arterial diseases is the profunda femoris artery (PFA) [10]. Last but not least, an introducer sheath is often used as a DPC even though it has not been designed, assessed, or validated for this indication. As a result, the efficacity of DPCs in preventing ischemic complications is still debated [5]. Some studies even suggest that a DPC could fail to prevent ALI, with Tanaka et al. reporting ALI in 12% of VA-ECMO patients [11], and Vallabhajosyula et al. reporting ALI in 26% of VA-ECMO patients who underwent percutaneous placement of the DPC [9].

We hypothesized that ALI still occurs under VA-ECMO because the DPC fails to provide adequate perfusion to the cannulated lower limb. The aim of this study was to analyze the impact of the arterial cannula diameter, the DPC diameter, and the common femoral artery (CFA) section limitation on the perfusion of the ipsilateral lower limb. To do so, a computational fluid dynamics (CFD) study was performed on a patient-specific arterial model to compare the flow distribution obtained in the lower limbs with several combinations of the three physical parameters of interest.

## 2. Materials and Methods

### 2.1. Study Design

Several combinations of arterial cannula diameters of 21 Fr, 19 Fr, 17 Fr, and 15 Fr with DPC diameters of 10 Fr, 8 Fr, and 6 Fr were studied. In our patient-specific arterial model, the 21 Fr arterial cannula limited the section of the CFA by 90%, while the other diameters of the arterial cannula limited the section by 75% for the 19 Fr, 57% for the 17 Fr, and 46% for the 15 Fr. To investigate the influence of the arterial section limitation by the arterial cannula on the perfusion of the lower limb and to account for inter-patient variability, a first series of simulations was done with the actual outer diameter. Then, a second series of simulations was performed with the outer diameter being increased to 21 Fr, in order to represent conditions where the arterial cannula is associated with a section limitation of the CFA because the patient has smaller arteries or because the CFA spasms around the arterial cannula.

### 2.2. Endpoints

The primary endpoint was the Ipsilateral/Contralateral Distal Flow Index, defined as the ratio of the distal flow of the CFA on the ipsilateral limb and the contralateral limb and obtained with the following formula:

$$Ipsilateral/Contralateral\;Distal\;Flow\;Index=\frac{Q_{ipsilateral\;CFA}}{Q_{contralateral\;CFA}}$$

A normal *Ipsilateral/Contralateral Distal Flow Index* is therefore 1. As the flow was measured in distal arteries only, the flow in the CFA was defined as the sum of flows in the SFA and PFA with the following formula:

$$Q_{CFA}=Q_{SFA}+Q_{PFA}$$

Secondary endpoints included the Absolute Distal Flow and the Distal/Proximal Flow Index.

The Absolute Distal Flow was calculated with the following formula:

$$Q_{Absolute\;Distal\;Flow}=Q_{ipsilateral\;SFA}+Q_{ipsilateral\;PFA}$$

The Distal/Proximal Flow Index was calculated with the following formula:

$$Distal/Proximal\;Flow\;Index=\frac{Q_{ipsilateral\;CFA}}{Q_{return\;cannula\;+\;Q_{ipsilateral\;CFA}}}$$

with

$$Q_{return\;cannula}=Q_{aorta}+Q_{ipsilateral\;internal\;iliac\;artery}+Q_{contralateral\;internal\;iliac\;artery}+Q_{contralateral\;CFA}$$

### 2.3. Arterial Model

**Figure 1:**
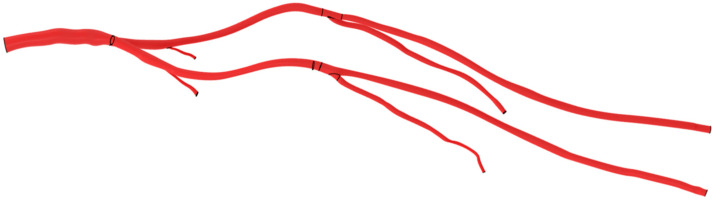
Patient-specific CAD model of the aorta and lower arterial branches. Black lines represent arterial bifurcations and CAD extremities.

An arterial model was obtained from the computed tomography (CT) angiography of a healthy adult woman whose anatomy was deemed representative of the overall adult population. The geometry of the arterial tree from the abdominal aorta to the PFA and SFA in both lower limbs was extracted manually using the CRIMSON software (Cardiovascular Integrated Modelling & Simulation, CRIMSON GUI 2022.02.04 and Flowsolver 1.5.3) [12]. Firstly, the centers of each artery were marked at regular intervals. Then, the contours of the arteries were drawn around each point. From this information, the software interpolates the geometry to generate a 3D computer-assisted design (CAD) segmentation. The diameters of the aorta, common iliac, external iliac, common femoral, and superficial femoral arteries were 13.1 mm, 7.8 mm, 7.2 mm, 6.9 mm, and 5.3 mm, respectively (Figure 1).

### 2.4. Cannula and DPC Models

**Figure 2:**
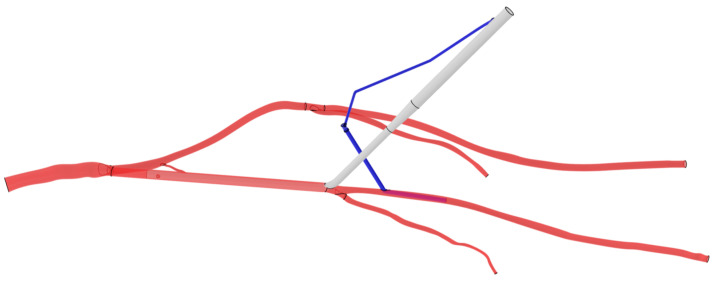
CAD model of the arterial cannula and the distal perfusion in the arterial system. The arterial cannula is white, the DPC is blue and the arteries are red. Black lines represent arterial bifurcations and CAD extremities.

The arterial cannula and DPC were drawn with the CATIA software (3DEXPERIENCE Platform, Dassault System, Vélizy-Villacoublay, France). The devices’ geometries are reproductions of commercially available devices. When an arterial cannula is inserted in the femoral artery, the artery straightens along the cannula. So, to simplify the model, the common femoral artery on the cannulated side has been replaced by a cylinder of equivalent inner and outer diameter. When the outer diameter is 21 Fr, the cannula reduces the internal section of the artery, and a hypothesis of no blood flow in the internal iliac artery on the cannulated side is made. Indeed, since the tip of the arterial cannula is higher than the internal iliac artery, it means that in conditions of a reduced section, the walls of the arterial cannula are against the primitive iliac bifurcation and prevent the blood from going toward the internal iliac artery. So, the ipsilateral internal iliac artery is not included in models where the outer diameter of the cannula is 21 Fr. The cannulas and DPCs were connected by a tube of 3 mm diameter and 20 cm length. The cannula is always inserted 14.4 cm into the common femoral artery, just before the bifurcation between the superficial femoral artery and the Profunda Femoris Artery. The DPC was placed in the superficial femoral artery with an entry point 4 cm away from the cannula (Figure 2).

### 2.5. CFD

The CFD analyses were performed on the SIMULIA Software (3DEXPERIENCE Platform, Dassault System, Vélizy-Villacoublay, France). The simulations were performed in a steady state of a full flow of the VA-ECMO with no residual pulsatility from the heart. The simulations were assumed to be carried out once the ECMO flowrate was stabilized and constant and did not account for pulsatility and deformation of the arterial walls. The arterial walls were therefore assumed to be rigid boundaries with no slip. As blood is considered at a constant temperature, with stable hematocrit, and in arteries larger than 1 mm, the blood was considered a Newtonian fluid with a constant viscosity [13,14,15]. As commonly stated, the blood was modeled with a density of 1060 kg/m^3^ and a dynamic viscosity of 4 × 10^−3^ Pa·s [16,17]. No flow was coming from the heart of the patient, and a constant flowrate of 5 L/min from the cannula was defined as the input, which corresponded to the required amount of perfusion to assure homeostasis in a patient of 70 kg [14]. The mean arterial pressure of 70 mmHg was defined at the pressure outlet of each peripheral artery [16]. According to Nakamura et al., during a cardiopulmonary bypass, the difference in pressure between the peripheral arterial pressure and the arterial pressure in the aorta is 11.6 mmHg [18]. A pressure of 81.6 mmHg was then defined at the outlet of the aorta.

### 2.6. Meshing

The dimensions of the elements for the meshing were defined according to a grid independence study on the combination of a 21 Fr arterial cannula with a 8 Fr DPC. The number of elements depends on the following parameters: the geometry of the meshing, the minimal and maximal sizes of the elements, and the number of layers and their thickness. When fixing all the parameters except the minimum element size, changing this value from 0.2 mm (16,241,383 elements) to 0.15 mm (31,058,112 elements) increases the cannula outlet pressure by 0.7%, which is considered a negligible variation. For all the simulations, the minimum element size was then set to 0.2 mm. The meshing was set with 3 boundary layers of 0.02 mm and majorly composed of hexahedral elements of sizes between 0.2 and 4 mm. Hence, the number of elements for all the simulations was between 1.53 × 10^7^ and 1.66 × 10^7^.

### 2.7. Statistical Analysis

Categorical variables were presented as numbers and proportions and compared with Chi-squared or Fisher’s exact test when appropriate. Continuous variables were reported as the median [interquartile range]. When the standard deviation of a variable was proportional to the absolute factor level, we applied a logarithmic transformation. Factors associated with primary and secondary endpoints were studied in a univariate analysis using linear regression, ANOVA, and the Williams test. Then, a multivariable analysis was performed using a linear regression model including prespecified variables of interest and interaction terms. All the data analyses were conducted with two-sided tests. A *p*-value less than 0.05 was considered significant. Statistical analyses were conducted using R (version 4.3.1, The R Statistical Software, R Foundation, Vienna, Austria).

## 3. Results

### 3.1. Flow Direction

**Figure 3:**
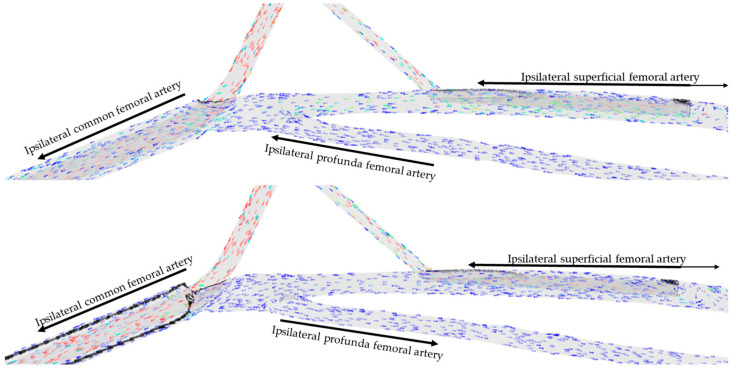
Direction of blood flow at the femoral level for two percentages of section limitation (46% up, 90% down) for an arterial cannula of 15 Fr with a distal perfusion cannula of 8 Fr. Arrows represent the direction of flow in each artery. Colors represent flow velocity, slow in blue, fast in red.

Visual examination of the simulations showed that blood circulating in the distal SFA was directed toward the lower limb in all but one configuration tested. Blood circulating in the proximal SFA above the DPC, in the PFA, and in the CFA was directed toward the lower limb if the arterial cannula limited the section of the artery by 90%, and toward the aorta along the arterial cannula if the arterial cannula limitation of the section was less than 90% (Figure 3). This finding was confirmed whatever the diameter of the DPC and can even result in a negative flow from the lower limb perspective in the case of a 15 Fr arterial cannula with a limitation of the section by 46% associated with a 6 Fr DPC.

**Figure 4:**
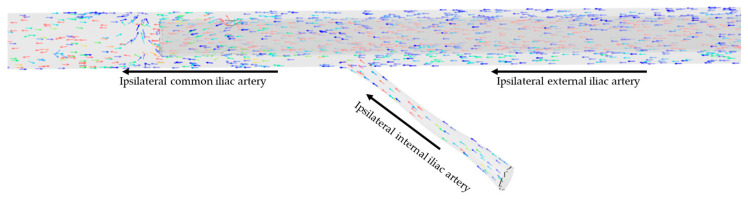
Direction of blood flow at the internal iliac level with an arterial cannula of 15 Fr, a DPC of 8 Fr, and a 46% limitation of the arterial section. Arrows represent the direction of flow in each artery. Colors represent flow velocity, slow in blue, fast in red.

Similarly, a visual examination of the simulations showed that blood circulating in the ipsilateral internal iliac artery, considered null in the case of limitation of the section of the artery by 90%, became negative when considering an arterial cannula with a limitation of the arterial section below 90%, with the blood directed toward the aorta along the arterial cannula (Figure 4).

### 3.2. Ipsilateral/Contralateral Distal Flow Index

The conditions and endpoints of each simulation are presented in Table 1. A desirable Ipsilateral/Contralateral Distal Flow Index approximating 1 was achieved in only 2 cases out of the 21 configurations tested. Ipsilateral/Contralateral Distal Flow Indexes of 0.94 and 1.03 were achieved with a 17 Fr arterial cannula with a section limitation of 90% and associated with 8 Fr and 10 Fr DPCs, respectively. Under these conditions, the flows in the ipsilateral and contralateral CFAs were similar. Larger arterial cannulas led to a decreased Ipsilateral/Contralateral Distal Flow Index, smaller arterial cannulas with arterial section limitation of 90% led to increased Ipsilateral/Contralateral Distal Flow Indexes, and smaller arterial cannulas with arterial section limitation below 90% led to decreased Ipsilateral/Contralateral Distal Flow Indexes, which turned negative in the case of the 15 Fr arterial cannula with 46% of arterial section limitation and 6 Fr DPC.

Specifically, for small arterial cannula with arterial section limitation below 90% combined with a small DPC, the flow into the PFA was negative, giving a median flow of −4 [−23–1] mL/min into the PFA for a percentage of section limitation below 90%, compared with 2 [1.75–3.25] mL/min when the percentage of section limitation was equal to 90%. In the SFA, the median flow was 76 [56–87] mL/min for a percentage of section limitation below 90% and 85 [64–121] mL/min for a percentage of section limitation of 90%. On the other hand, flow in the contralateral primitive iliac artery was measured in all the simulations at 131 [128–132] mL/min and did not vary significantly with changes in the percentage of section limitation and the diameter of the cannula. Thus, as the arterial cannula diameter, DPC diameter, and percentage of section limitation decreased, the Ipsilateral/Contralateral Distal Flow Index decreased, as well.

### 3.3. Absolute Distal Flow and Distal/Proximal Flow Index

For all the simulations, the inlet flowrate of the VA-ECMO was fixed to 5 L/min, so Absolute Distal Flow and Distal/Proximal Flow Index were linearly correlated. The highest Distal/Proximal Flow Index was obtained with a 15 Fr arterial cannula with an arterial section limitation of 90% combined with a 10 Fr DPC. Under these conditions, the Absolute Distal Flow was estimated to be 202 mL/min, corresponding to 4.03% of the total flow of the VA-ECMO. The lowest Distal/Proximal Flow Index was obtained with a 15 Fr arterial cannula with a section limitation below 90% combined with a 6 Fr DPC and was equal to −0.35%. This flowrate was negative because the blood flow circulating from the SFA and the PFA toward the aorta was higher than the blood flow coming from the DPC toward the distal SFA.

### 3.4. Physical Determinants of Lower Limb Perfusion under VA-ECMO

**Figure 5:**
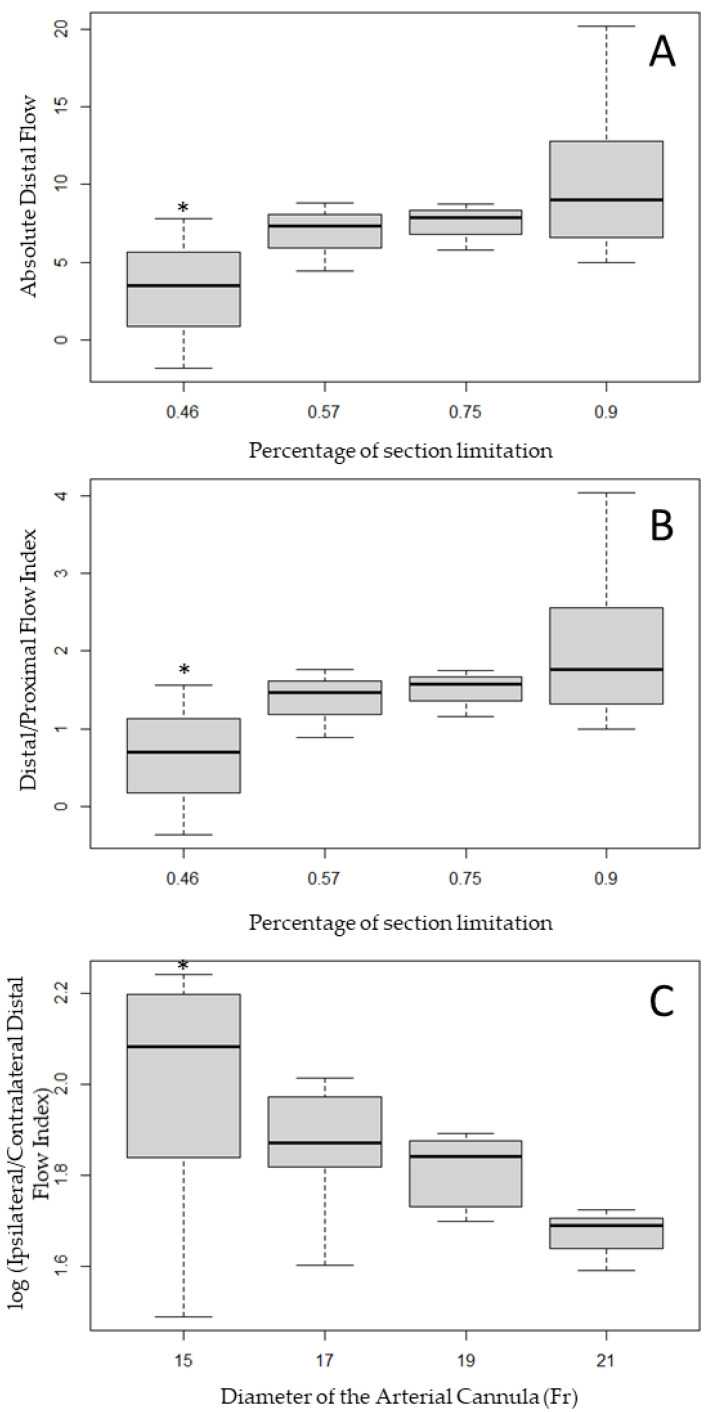
Distribution of the Absolute Distal Flow (**A**) and Distal/Proximal Flow Index (**B**) in a function of the percentage of section limitation and distribution of the log (Ipsilateral/Contralateral Distal Flow Index) in a function of the diameter of the arterial cannula (**C**). * corresponds to a *p*-value < 0.05 when compared with the distribution obtained percentage of section limitation of 0.90 or an arterial cannula diameter of 21 Fr.

Linear regressions were performed on all the combinations of explanatory variables and endpoints. Three combinations showed a significant linear relation, and their correlations were confirmed using ANOVA. The Absolute Distal Flow (linear regression, *p* = 0.013; ANOVA, *p* = 0.028) and the Distal/Proximal Flow Index (linear regression, *p* = 0.012; ANOVA, *p* = 0.013) were significantly correlated with the percentage of section limitation, and both secondary endpoints increased significantly when the percentage of section limitation increased (Figure 5A,B).

The Ipsilateral/Contralateral Distal Flow Index was significantly correlated with the diameter of the arterial cannula, with higher diameters associated with lower perfusion of the ipsilateral limb (Figure 5C, linear regression, *p* = 0.029; ANOVA, *p* = 0.012). Standardized studentized residuals showed that three configurations were outliers and all were related to the 15 Fr arterial cannula: (i) when considered as limiting the section by 46% and combined with a DPC of 6 Fr leading to a negative Absolute Distal Flow; or (ii) and (iii) when considered as limiting the section by 90% and combined with a DPC of 8 and 10 Fr, leading to higher flow in the ipsilateral than in the contralateral limb.

The perfusion of the lower limb was therefore significantly associated with the size of the arterial cannula compared with the CFA diameter, and the ratio between the ipsilateral and contralateral distal flow was associated with the size of the arterial cannula. On one hand, the median Absolute Distal Flow and the median Distal/Proximal Flow Index increased when the percentage of section limitation increased, i.e., when the external diameter of the arterial cannula gets closer to the internal diameter of the CFA. On the other hand, when the internal diameter of the arterial cannula increased, the median Ipsilateral/Contralateral Distal Flow Index decreased.

The means are monotonically ordered, so the last test performed on the three combinations was the Williams test. A diameter of the arterial cannula of 15 Fr resulted in an Ipsilateral/Contralateral Distal Flow Index significantly lower than with an arterial cannula of 21 Fr. When compared with a percentage of section limitation of 90%, having a percentage of section limitation equal to or lower than 46% resulted in a significant difference in the mean Absolute Distal Flow and the mean Distal/Proximal Flow Index (*p* < 0.05). The results of the ANOVA and the Williams test show that the most unfavorable conditions for distal perfusion are reached when the section’s area of the arterial cannula is lower than half the section’s area of the CFA.

In the multivariable analysis, the Ipsilateral/Controlateral Distal Flow Index was associated with the diameter of the DPC (*p* = 0.01884), but also with the diameter of the arterial cannula (*p* = 0.00739) and the percentage of section limitation (*p* = 0.00216). The interaction of the percentage of section limitation with the diameter of the arterial cannula was highly significant (*p* = 0.00478). To obtain adequate perfusion of the cannulated lower limb during VA-ECMO, the important parameters include the diameter of the DPC, but also the diameter of the arterial cannula, and the percentage of arterial section limitation.

## 4. Discussion

Investigating the efficacy of a DPC to maintain adequate blood flow toward the canulated lower limb during VA-ECMO, we studied four diameters of arterial cannula, three diameters of DPC, and two percentages of arterial section limitation, and found that symmetric perfusion of the lower limbs was achieved in only 2 out of the 21 configurations tested, specifically when the arterial cannula had a diameter of 17 Fr, with a limitation of the arterial section of 90%, and was associated with 8 Fr and 10 Fr DPCs. Arterial cannulas with section limitation below 90% could result in decreased perfusion of the cannulated lower limb through a stealing effect, redirecting blood from the CFA, PFA, and proximal SFA toward the aorta despite the presence of the DPC. Multivariable analysis revealed that the perfusion of the cannulated lower limb was correlated with the diameter of the DPC, but also with the diameter of the arterial cannula and the percentage of arterial section limitation.

### 4.1. ALI under ECMO

The reported incidence of ALI under VA-ECMO varies from one study to another, ranging from 11% to 52%. A prospective trial found an incidence of critical limb ischemia of 38% in patients with refractory cardiogenic shock supported with VA-ECMO [19]. ALI can lead to further complications and negatively affect patients’ survival [3]. The use of a DPC aims at avoiding ALI, but its efficiency has not yet been proven, and up to 26% of patients under VA-ECMO experience ALI despite the use of a DPC [9]. As a consequence, the prevention of ALI during VA-ECMO is still debated. To prevent and treat ALI, Hu et al. recommend a “4S” scheme [20] that includes (i) a puncture site for the arterial cannula on the CFA; (ii) an optimal sizing of the arterial cannula that should be inferior to 80% of the diameter of the CFA and the smallest size possible able to ensure the targeted flowrate; (iii) a systematic evaluation of limb perfusion; and (iv) a salvage intervention with the placement of a DPC as soon as limb ischemia is diagnosed. Conversely, the ELSO guidelines advocate for the systematic and early placement of a DPC in order to prevent ALI [6].

In practice, it is often preferred to use a smaller arterial cannula, with the idea that the blood can flow around the cannula from the aorta toward the cannulated CFA, and to add a DPC once the arterial cannula has been placed [9,21]. Currently, the only device intended to be used as a DPC and available on the market is the CruraSave femoral perfusion set (Free Life Medical GmbH, Aachen, Germany) [21,22]. However, an extensive literature review revealed that most of the time, DPCs are introducer sheaths designed to give temporary arterial access. Those devices are not designed to deliver a prolonged blood flow, they are not antithrombogenic, and the connection between the sheath and the cannula is a three-way stopcock that leads to significant flow limitation [21]. Introducer sheaths do not allow an optimal flow to the ipsilateral limb, and a thrombus can be formed inside the DPC. This suboptimal strategy is thus still associated with high rates of ALI, and most studies have not been able to prove that the size of the cannula or the adjunction of a DPC is associated with a significant decrease in the occurrence of this threatening complication.

### 4.2. CFD

To elucidate the physical determinants of lower limb perfusion during VA-ECMO, we defined a set of CFD simulations that combine a patient-specific arterial model, four sizes of arterial cannula, three sizes of DPC, and two percentages of arterial section limitation. Image-based arterial models are constructed with CAD software from parameters segmented from images derived from CT angiographies or 4D-MRI [16,17], thus allowing detailed geometrical features. The model represents only one individual, but we have chosen a patient representative of a population with no arterial disease. Furthermore, after setting the diameter of the arterial cannula and the diameter of the DPC, we set a third parameter representing the percentage of section limitation of the CFA by the arterial cannula, allowing the generalization of our findings to patients with different CFA diameters.

After the choice of the arterial model, the parameters of the CFD study should be thoroughly established. Blood is a non-Newtonian fluid because of its cellular content. Its viscosity depends on the temperature, the hematocrit, and the shear rate [13]. There are different ways to model the blood’s viscosity, the most common being the Carreau–Yasuda model [14,15], the power law model, and the Quemada model. In vessels with a diameter greater than 1 mm and media with high shear rates (higher than 100 s^−1^) [13], it can be assumed that blood behaves as a Newtonian fluid. In our study, the blood was considered at a constant temperature, with stable hematocrit, in arteries large enough so that the interactions between the blood cells and the arterial walls are negligible. The blood was then considered a Newtonian fluid with a constant viscosity, as in many other CFD studies focusing on ECMO [16,23,24,25].

### 4.3. Medical Technology

Interestingly, the first part of our study confirmed the findings of Bongert et al. [26], who used a CFD model to study the perfusion of a cannulated leg without DPC. Both studies found an antegrade flow toward the non-cannulated side generally greater than that toward the cannulated side, and a negative flow from the ipsilateral superficial femoral artery toward the aorta. In the study by Bongert et al., an analysis of pressure showed the induction of zones of negative pressure close to the tip of the arterial cannula, consistent with the Bernoulli principle [26]. In our study, the adjunction of the DPC in the simulations revealed that the antegrade blood flow associated with the DPC is counterbalanced by the stealing effect of the arterial cannula, with blood directing from the CFA, PFA, and proximal SFA toward the aorta. This stealing effect can even neutralize the flow of the DPC in the case of the 15 Fr arterial cannula with section limitation by 46% and 6 Fr DPC.

Our study is also interesting because it is the first study to identify the degree of section limitation of the CFA by the arterial cannula as an independent factor associated with the blood flow of the cannulated lower limb in multivariable analysis. The results of the simulations clearly show that when using a DPC routinely, better distal perfusion is obtained using an arterial cannula whose section is equal to 90% of the CFA section than when it is lower than 80%. This way, most of the blood perfused from the DPC is directed toward the distal SFA, and a portion of the DPC flow is even directed toward the PFA. To minimize the stealing effect from the aorta and to optimize the blood flow toward the cannulated limb, our results suggest that the arterial cannula should reduce the section by at least 90%.

A sizing of the femoral arteries is therefore important to choose the adequate arterial cannula. Additionally, it is of tremendous importance to perfuse the cannulated limb at the time of arterial cannula placement, as a decrease in perfusion in the lower limb of more than 15% for more than 4 min is known to be associated with clinically meaningful leg ischemia [27]. Ideally, it would also be very important to obtain perfusion of the CFA to perfuse both the PFA and the SFA, as the PFA has been clearly shown to be the main artery of the lower limb in patients with arterial diseases [10] and is only sparsely perfused with the DPC. Physicians involved in ECMO implantation should therefore be able to perform a thorough assessment of patient vessels and implantation sites, which might involve ultrasound capabilities in the emergency setting.

### 4.4. Study Limitations

This study is a work of simulations. Thus, several hypotheses were made to simplify the conditions of the simulations, especially concerning the constant outlet pressure and the steady state, which can impact the results. We have chosen a normal anatomy, thus limiting the application of our study to patients without peripheral arterial disease. However, all the hypotheses were carefully based on the existing literature. All the combinations were studied, but some would not be used in practice. Of note is the fact that the perfusion of the cannulated limb and its tolerance to a certain degree of ischemia are influenced by the arterial flow studied here, but also by blood content, oxygen saturation, venous pressure, and individual factors that have not been taken into account in this study [28].

## 5. Conclusions

Investigating the efficacy of a DPC to maintain adequate blood flow to the cannulated lower limb during VA-ECMO, we studied four diameters of arterial cannula, three diameters of DPC, and two percentages of arterial section limitation, and found that adequate perfusion of the cannulated limb was achieved in only 2 out of the 21 configurations tested and was influenced by the diameter of the DPC, but also by the diameter of the arterial cannula and the percentage of arterial section limitation.

In most of the cases, the current system combining unsized arterial cannulas and non-specific DPCs is not sufficient to provide an adequate perfusion of the cannulated lower limb, urging the need for innovative strategies to efficiently prevent ALI during peripheral VA-ECMO. Additional studies will seek to develop a systematic, immediate, adequately sized, and CFA-centered perfusion of the lower limb downstream of the VA-ECMO arterial cannula.

## Figures and Tables

**Table 1:** Physical parameters and endpoints of each simulation.

Cannula (Fr)	Percentage of Section Limitation	Distal Perfusion (Fr)	Ipsilateral/Contralateral Distal Flow Index	Distal/Proximal Flow Index (%)	Absolute Distal Flow (mL/min)
21	90%	6	0.39	0.99	50
		8	0.49	1.26	63
		10	0.53	1.37	68
19	90%	6	0.54	1.26	63
		8	0.71	1.68	84
		10	0.78	1.85	92
	75%	6	0.50	1.15	58
		8	0.68	1.58	79
		10	0.75	1.73	87
17	90%	6	0.70	1.59	80
		8	0.94	2.13	107
		10	1.03	2.36	118
	57%	6	0.40	0.89	45
		8	0.66	1.47	73
		10	0.79	1.76	88
15	90%	6	1.21	2.77	138
		8	1.58	3.67	183
		10	1.74	4.03	202
	46%	6	−0.16	−0.35	−18
		8	0.31	0.70	35
		10	0.69	1.56	78

## Data Availability

Data can be available upon reasonable request to the authors.
